# Spatio-Temporal Saliency Perception via Hypercomplex Frequency Spectral Contrast

**DOI:** 10.3390/s130303409

**Published:** 2013-03-12

**Authors:** Ce Li, Jianru Xue, Nanning Zheng, Xuguang Lan, Zhiqiang Tian

**Affiliations:** Institute of Artificial Intelligence and Robotics, Xi'an Jiaotong University, Xi'an 710049, China; E-Mails: nnzheng@mail.xjtu.edu.cn (N.Z.); xglan@mail.xjtu.edu.cn (X.L.); tianzq@gmail.com (Z.T.)

**Keywords:** spatio-temporal, salient object, hypercomplex, visual attention, visual perception, spectral contrast

## Abstract

Salient object perception is the process of sensing the salient information from the spatio-temporal visual scenes, which is a rapid pre-attention mechanism for the target location in a visual smart sensor. In recent decades, many successful models of visual saliency perception have been proposed to simulate the pre-attention behavior. Since most of the methods usually need some *ad hoc* parameters or high-cost preprocessing, they are difficult to rapidly detect salient object or be implemented by computing parallelism in a smart sensor. In this paper, we propose a novel spatio-temporal saliency perception method based on spatio-temporal hypercomplex spectral contrast (HSC). Firstly, the proposed HSC algorithm represent the features in the HSV (hue, saturation and value) color space and features of motion by a hypercomplex number. Secondly, the spatio-temporal salient objects are efficiently detected by hypercomplex Fourier spectral contrast in parallel. Finally, our saliency perception model also incorporates with the non-uniform sampling, which is a common phenomenon of human vision that directs visual attention to the logarithmic center of the image/video in natural scenes. The experimental results on the public saliency perception datasets demonstrate the effectiveness of the proposed approach compared to eleven state-of-the-art approaches. In addition, we extend the proposed model to moving object extraction in dynamic scenes, and the proposed algorithm is superior to the traditional algorithms.

## 1. Introduction

Visual attention is an important cognitive mechanism of human survival. Humans have the capability of rapidly focusing on potential objects in a cluttered visual world based on selective visual attention, which has been studied in physiology, psychology, neural systems and computer vision for a long time [1]. The salient objects or regions often contain important semantic content, which could be applied to visual semantic analysis, such as visual servoing of autonomous mobile robots [2], motion object detection [3], image/video segmentation [4,5], scene recognition [6], smart video surveillance [7], object recognition [8] and image compression [9].

Visual saliency is a perceptual quality that makes an object distinguishable to its neighbors and, thus, captures our attention. Existing saliency approaches can be divided into two categories: task-driven attention (top-down) and data-driven attention (bottom-up). The top-down approach is a result of long-term visual simulation with prior knowledge. It is slow and task driven [10–12]. The bottom-up approach is based on low-level visual features simulating the formation of the short-term visual attention. In contrast to the top-down method, the bottom-up approach is rapid and without prior knowledge. It is a data contrast-driven mechanism in pre-attentive vision for salient objects without task [1,13–28]. In this paper, we only focus on the bottom-up approach.

Compared with task-driven visual attention, which is not clear yet, data-driven visual attention is studied extensively. Since the well-known feature integration theory (FIT) was published by A. Treisman and G. Gelade [29], there has been a growing interest in data-driven attention. Among these models, Itti and Koch's model [13] is the most famous one. They detected a saliency map by the center-surround operator and normalizing a set of low-level features. Based on the Itti's model, N. Bruce *et al.* proposed an information maximization detection model [14]. Liu and Zheng modeled visual attention by a CRF (conditional random field) learning algorithm [1]. Goferman introduced context information in salient object detection [25]. However, most of the methods usually need some *ad hoc* parameters or high-cost preprocessing, and they have difficulty in rapidly detecting a salient object.

Recently, visual saliency perception in the frequency domain has become popular. Hou [26] proposed a fast Fourier transform spectral residual analysis algorithm for image saliency detection. In this method, amplitude spectral residual is considered as an important factor to stimulate visual attention. Furthermore, Guo [27] proposed a saliency detection algorithm by using the phase spectrum of the quaternion Fourier transform. Achanta [28] gave a simple and effective salient region detection solution by the frequency-tuned method. However, for saliency perception, the problem is, which one is more important, the amplitude spectrum or the phase spectrum? Meanwhile, how does one implement visual saliency perception processing in computing parallelism? In this paper, we argue that the phase spectrum contains image structure information, and the amplitude spectrum carries the visual perception magnitude information. Based on the theories of [29,30] and the saliency detection methods of [1,13,25–28], we propose a computing parallelism algorithm named HSC, considering both amplitude spectrum and phase spectrum in a multi-scale hypercomplex of HSV (hue, saturation and value) color space and motion feature (see Section 3 for details):

1. In the frequency domain, amplitude spectrum and phase spectrum are both significant for saliency detection. Either one of them could not reconstruct a whole saliency map in the frequency domain.
2. A saliency map is the product of various visual features of comprehensive stimulation. United multi-feature vector expression would be an efficient computation method. In particular, the spatio-temporal image sequence of significance is the result of dynamic and static characteristics of integrated stimulus.
3. Spatio-temporal saliency perception is a rapid processing result of multi-features contrasting in parallel in multi-scales.
4. The position of a pixel is important to saliency detection in an image, since people tend to focus their attention on some specific areas.

The remainder of this paper is organized as follows: in Section 2, we summarize and analyze existing algorithms. Section 3 gives the details of our visual saliency perception model, including spatio-temporal hypercomplex spectral contrast computation, a log-polar bias sampling strategy and saliency map computation. Section 4 presents and discusses the experimental results and evaluations for our model by comparing the proposed approach with other state-of-the-art methods on more than 1,000 natural and psychological images. In Section 5, we discuss the difference between and the proposed algorithm with other related methods. Section 6 explores the application of the proposed approach in moving object extraction in dynamic scenes. Finally, conclusions and future works are given in Section 7.

## 2. Related Work

In many classical visual perception applications, the spectrum of an image has many applications, such as denoising, enhancement, compression and matching. The frequency domain transform and human visual perception mechanism also have a close relationship. In Itti's saliency model [13], the Gabor wavelet frequency transform is used to get orientation information in the image. From Piotrowski's [31] and Oppenheim's [32] viewpoint, we believe that the phase spectrum contains image structure information, and the amplitude spectrum carries the visual perception magnitude information. Frequency transform has been widely used in various approaches of saliency detection, such as Fourier transform (FT) [20,26], quaternion Fourier transform (QFT) [24,27,33–35], discrete cosine transform (DCT) [36,37] and quaternion discrete cosine transform (QDCT) [38,39]. The salient object is highlighted in the visual attribute synthesis difference. The kind of saliency feature has holistic and multi-scale contrast (global/local contrast) in visual perceptual stimulus. From the frequency domain spectrum of information, feature space in parallel processing and global/local contrast in multi-scale, we analyze these three aspects of saliency perception models by frequency transform in detail below, as shown.

### Using the frequency domain spectrum of information

**Figure 1.**
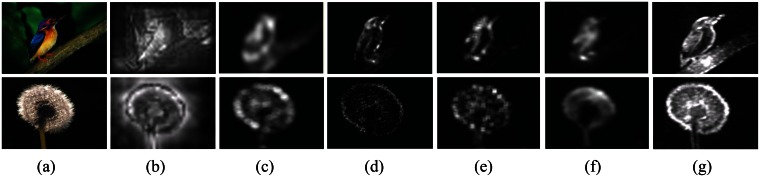
Comparison of five saliency perception algorithms, (**a**) Raw images, Bird: color higher contrast, Dandelion: texture higher contrast; (**b**) saliency maps of Itti's method [13]; (**c**) saliency maps of Hou's method (spectral residual (SR)) [26]; (**d**) saliency maps of Guo's method phase spectrum of quaternion Fourier transform (PQFT) [27]; (**e**) Saliency maps of quaternion discrete cosine transform (QDCT) [38]; (**f**) saliency maps of HFT [34]; (**g**) saliency maps by the proposed saliency perception method (hypercomplex spatiotemporal spectral contrast (HSC)).

Generally, the key of bottom-up saliency detection is extracting and integrating a variety of visual properties from contrast differences. Saliency detection based on the frequency domain model is no exception. These kind of models can be divided into two categories: amplitude-based and phase-based. Hou [26] designed a simple and fast saliency detection approach by an amplitude spectral residual (SR). In this method, Hou assumed that the image information is made up of two parts: innovation and prior knowledge. The author believed that the statistical singularities in the amplitude spectrum may be responsible for anomalous regions in the image, where salient objects pop up. However, Guo [27] believed that the phase spectrum is a key factor to visual saliency. The author pointed out that the salient region was often caused by the sudden change of phase (PQFT). Although these two methods have better preformation in salient objects detection, they are still insufficient. In fact, the frequency domain transforms and inverse transform implementation need the phase and amplitude of common information in order to accurately express that the image contains information. The amplitude information states the energy spectrum of mutations and the phase information states the textural change in an image. Based on the amplitude spectrum, the saliency detection method has a salient object pre-position ability, but the integrity of the object is poor. Other phase spectrum-based methods are sensitive to the boundary of a salient object. Too much emphasis on either one factor is not appropriate, as shown in the columns of Figure l(c,d). Based on this view, the proposed approach can detect more human vision salient objects in an image with a combination of amplitude and phase, as shown in the column of Figure 1(g).

### Parallel Computation of feature space

Multi-feature parallel computing cannot only speed up the computation, but also improve the performance of visual saliency perception. The saliency map of Itti [13] is generated based on the linear combination of normalized four saliency sub-maps: intensity, red-green color opponency, blue-yellow color opponency and orientation. The author in [20] discusses a method of saliency detection by a color conspicuous map and an orientation conspicuous map. In the approach of [26], the amplitude spectral residual is simply defined on a single feature of a gray image. These methods only obtained a saliency map from a single feature or simple combination of many sole features saliency sub-maps. The problem is that they do not take the internal relation and relevance into consideration. The authors in [35] pointed out that the approach of [20] will lose much information of salient objects by using simple or selecting simple color distribution or orientation distribution in an image. Different from above, our proposed saliency detection algorithm uses the color, intensity and texture information by hypercomplex number to obtain the final saliency map in parallel, as same as the methods of [27,35]. Compared with the results of the methods of [13,26], as shown in the columns of Figure 1(b,c), our saliency map has better subjective results, as shown Figure 1(g).

### Multi-scale global or local contrast

Visual scale space in general is important to saliency perception. The salient degree of objects is often the inconsistency of the scale space [40]. In [26,27], the saliency maps are obtained using an average filter. The study in [38,39] gets saliency map based on the salient value for each patch of quaternion discrete cosine transform (QDCT). As show in Figure 1(e), the approach's performance in salient object detection is limited. The local block frequency spectrum computing affects the detection results of this method. The work in [33,34] address a visual saliency perception approach by a scale-space analysis of the amplitude spectrum of natural images. This method [34] is able to predict salient regions on which people focus their attention. The authors assumed that the best saliency map would appear in a specific scale of an image, which has the maximum entropy among various scales of the image. The saliency map of optimal scale weakens the saliency perceived in the other scales of the salient object, as shown in Figure 1(f). In contrast, the proposed model uses global multi-scale contrast and incorporates the non-uniform sampling adopted by human vision, as shown in Figure 1(g).

Besides the saliency perception methods above, we assume that saliency perception can be taken as a filtering process, which is a performed in the frequency domain to filter out the average energy signal and retain various features of the integrated signal energy contrast larger spectral filtering process. So, we propose a novel spatio-temporal saliency perception method based on hypercomplex spatiotemporal spectral contrast (HSC). The contribution of this paper is two-fold. On the one hand, we propose a saliency perception method by hypercomplex spectral contrast in parallel. On the other hand, we introduce a log-polar bias sampling mechanism to imitate a non-uniform sampling of the human vision system. From Figure 1, we can see that the proposed method has a better performance on image detail detection, as a part of a bird's mouth (Figure 1(g)); our method is more sensitive to the texture, such as in Figure 1(g), of the dandelion's integrity. Different from our pre-work [24], we extend our pre-work from static saliency perception to spatio-temporal saliency perception. Additionally proving the robustness and effectiveness of our methods, we will extend the application to moving object extraction in this paper.

## 3. Our Approach

**Figure 2.**
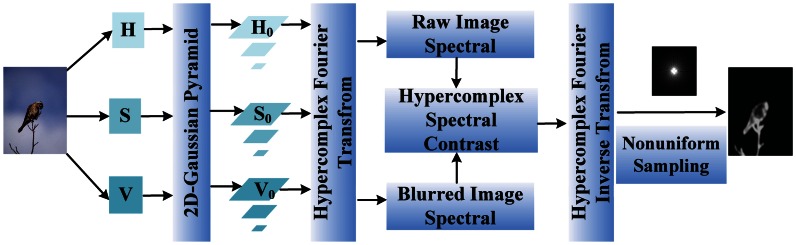
Overview of the HSC saliency perception framework.

In this section, we describe the proposed model in detail. The framework of our approach is illustrated in Figure 2. In this work, we compute a hypercomplex Fourier spectrum contrast of the amplitude and phase information using hypercomplex Fourier transform, respectively, in the multi-scale HSV color space. In this case, the saliency map could be produced using two hypercomplex spectral contrast maps at the same time by reconstruction and non-uniform sampling. The proposed HSC method mainly contains four steps:

***Step 1:*** Convert a raw image, **I**, to the HSV color space, and then **I** was blurred by 2D Gaussian on three level pyramids to eliminate fine texture details, as well as to average the energy of image **I**.
***Step 2:*** Represent image pixels by pure quaternion (hypercomplex) on HSV color space, then calculate the hypercomplex Fourier spectrum, which contains amplitude and phase information of the image by hypercomplex Fourier transform [41] in different scales.
***Step 3:*** Calculate the spectral contrast between the raw image and blurred image, and then, reconstruct these contrast maps using amplitude spectral and phase spectral under various scales of the raw image.
***Step 4:*** Normalize the reconstructed spectral contrast maps and use log-polar non-uniform sampling to obtain the final saliency map.

### 3.1. Hypercomplex of HSV Color Image

Quaternion is a kind of hypercomplex number. Color image pixels have inherently 3-D components, and they can be represented in quaternion form using pure quaternion [41]. A commonly used color space that corresponds more naturally to human perception is the HSV color space, which contains three components: hue, saturation and value. In this paper, each pixel of the raw image is represented by hypercomplex numbers (quaternion) consisting of HSV three-color components, which do not consider color opponent-component (RG or BY) and intensity, different from [27,40]. Thus, a hypercomplex number HSV image *q*(*x,y*) is defined as follows:

(1) q=Hi+Sj+Vk

where **i,j,k** satisfies **i**^2^ = **j**^2^ = **k**^2^ = −1, **i** ⊥ **j**, **j** ⊥ **k**, **i** ⊥ **k**, **k** = **ij**.

Based onp the definition above, the hypercomplex number HSV image *q's* pixel is given by pixel symplectic decomposition as:

(2) $$\begin{array}{l} q=f_{1}+f_{2}j\\ f_{1}=H\text{i}\\ f_{2}=S+V\text{i} \end{array}$$

### 3.2. Saliency Detection Using HSC

Usually, salient visual stimulus is often generated by strong contrast signals in the bottom-up model, which have a larger energy of spectrum. In another words, some strong spectral contrast of amplitude and phase are the main components in salient signals. In this paper, we calculate the amplitude spectrum and phase spectrum using the hypercomplex Fourier transform [41] of the HSV color image. Based on Equation (2), hypercomplex Fourier transform of the hypercomplex image, *q*, can be calculated by two complex Fourier transforms of the symplectic parts, such as:

(3) $$Q\left[u,\upsilon \right]=F_{1}\left[u,\upsilon \right]+F_{2}\left[u,\upsilon \right]j$$

We define each part of the forward and inverse hypercomplex Fourier Transform of Equation (3) in Equation (4):

(4) $$\begin{array}{c} F_{i}\left[u,\upsilon \right]=\frac{1}{\sqrt{MN}}\sum_{y=0}^{M-1}\sum_{x=0}^{N-1}e^{-j2\pi (\left(xu/N\right)+\left(y\upsilon /M\right)}f_{i}\left(x,y\right)\\ f_{i}\left[x,y\right]=\frac{1}{\sqrt{MN}}\sum_{u=0}^{M-1}\sum_{\upsilon =0}^{N-1}e^{j2\pi (\left(xu/N\right)+\left(y\upsilon /M\right)}F_{i}\left(u,\upsilon \right) \end{array}$$

where (*x,y*) is the spatial location of each pixel and (*u,v*) is the frequency domain. *M* and *N* are the height and width of the image.

Furthermore, using the above Equations (1–4), we completed the transform from *q* to *Q* in the hypercomplex frequency domain, which can be also defined in the polar form:

(5) $$Q=\left\|Q\right\|e^{\text{j}\phi }$$

where ‖*Q*‖, *φ* and ***j*** are the amplitude spectrum, phase spectrum and unit pure hypercomplex number, respectively.

In the next subsection, we first define the single-scale saliency of HSC. And then, we introduce the multi-scale analysis into the HSC method in order to refine the saliency detection result.

#### Single-scale saliency of HSC

First, we consider a single scale, *l*. Given an input raw image, **I**, we can obtain a blurred image **I***_b_* using a 2D-Gaussian filter (*σ* = 3). Using Equations (1–5), we calculate the amplitude spectrum $\left(\left\|Q_{I}^{l}\right\|,\left\|Q_{b}^{l}\right\|\right)$ and phase spectrum $\left(\phi_{I}^{l},\phi_{b}^{l}\right)$ of the raw image and blurred image in HSV color space, respectively, as follows:

(6) $$\begin{array}{c} Q_{I}^{l}=\left\|Q_{I}^{l}\right\|e^{j\phi_{I}^{l}}\\ Q_{b}^{l}=\left\|Q_{b}^{l}\right\|e^{j\phi_{b}^{l}} \end{array}$$

Then, our hypercomplex spectral contrast of each pixel, $CQ_{u,\upsilon }^{l}$, is obtained by:

(7) $$CQ_{\left(u,\upsilon \right)}^{l}=\text{log}({\left\|Q_{I(u,\upsilon )}^{l}\right\|}^{2}/{\left\|Q_{b(u,\upsilon )}^{l}\right\|}^{2})e^{j\phi_{I(u,\upsilon )}^{l}}$$

where *CQ*^1^ is the total of hypercomplex spectral contrast, the same as Equation (4) and (*u,v*) is the frequency domain, since, the blurred image has the average spectrum energy in hypercomplex frequency. Thus, the amplitude spectral contrast would represent the salient energy in the hypercomplex frequency domain. The phase spectral could represent the salient structure information in the hypercomplex frequency domain.

Hence, we use Equation (4) to obtain the reconstruction of $CQ_{\text{final}}^{l}$ as $cq_{I}^{l}$, represented as follows:

(8) $$cq_{I}^{l}=a+bi+cj+dk$$

Finally, our HSC saliency map, *S^l^*, at scale *l* is obtain by:

(9) $$S^{l}=f_{\text{Gaussian}}*{\left\|cq_{I}^{l}\right\|}^{2},\sigma =3$$

#### Multi-scale saliency of HSC

Given the existence of the multi-scale of human visual perception, we can obtain the set of multi-scale blurred images whose scales are *l* = {1,0.5,0.25}, in order to enhance our saliency detection result, using a 2D-Gaussian pyramid, Thus, the average of the HSC saliency map at various scales can be obtained as follows:

(10) $$S_{m}=\frac{1}{L}\sum_{l=1}^{L}S^{l}$$

### 3.3. Non-Uniform Sampling and Saliency Map

Our understanding of nature scenes is often from non-uniform observations in space or time. Usually, humans observe a natural image from its center. This means that the pixel's position is important to saliency detection in a image. From these above views, we design a simple method of logarithm center bias weight to simulate log-polar non-uniform sampling transform starting from the image center. We can calculate the log-center-distance, *D_log_*(*x,y*), between each pixel (*x,y*) and the image center. And then, we obtain the final saliency map as follows:

(11) $$SM_{\text{final}}(x,y)=S_{m}(x,y)/1+D_{\log}(x,y))$$

In the HSC algorithm, we use multi-scale hypercomplex spectral contrast and log-center-bias to implement the saliency detection. The proposed method is simple and effective, so it can be applied in digital media applications as a pre-processing approach.

### 3.4. Spatio-Temporal of HSC

**Figure 3.**
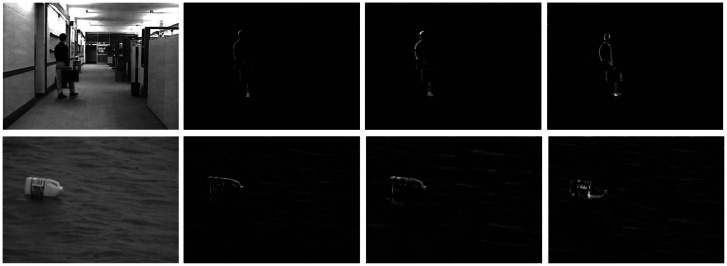
Compare with three kinds of frame difference methods. The first column is the monitor's image sequence of the static background. The second column is the boat's image sequence of dynamic background. From left to right: original video, results of two frames' difference, three frames' difference and the proposed method.

Generally, the visual attention of humans is more sensitive to moving objects than static objects. The classic spatio-temporal saliency detection methods calculated the temporal and spatial attention models separately. It is necessary to collaborate these two models in a meaningful way to produce the final spatio-temporal saliency maps by one or two weights for the temporal and spatial attention models, such as [42,43]. Although such methods often give a better results of saliency detection, the *ad hoc* parameters are difficult to adapt to a variety of video data. In this paper, we extend the above saliency detection model to spatio-temporal field. We add the multi-scale motion cue (as show in Equation (12)) to the HSC model described above. For the *t* time frame *I_t_*(*x, y*) of video intensity feature, Equation (12) is a simple motion estimation with the difference of using five frames. In contrast, Equation (12) has better noise immunity than two frames' difference and three frames' difference, as show in Figure 3.

(12) $$M_{t}(x,y)=\frac{1}{8}\left\{I_{t-2}(x,y)-4I_{t-1}(x,y)-I_{t}(x,y)+4I_{t+1}(x,y)-I_{t+2}(x,y)\right\}$$

After introducing the motion cue, Equation (1) can be reformed into Equation (13). Then, we can get a new spatio-temporal hypercomplex using spatial cue and motion cue:

(13) $$q_{t}=M_{t}+H_{t}i+S_{t}j+V_{t}k$$

(14) $$\begin{array}{l} q_{t}=f_{1t}+f_{2t}\text{j}\\ f_{1t}=M_{t}+H_{t}\text{i}\\ f_{2t}=S_{t}+V_{t}\text{i} \end{array}$$

Then, according to the Equation (14), we can compute a spatio-temporal saliency map by the above HSC Equations (3–11).

## 4. Experimental Validation

In this paper, we evaluate the proposed method in three groups of experiments: psychological pattern response, static saliency detection in natural images and saliency detection in dynamic scenes, respectively. In psychological pattern response, the psychological stimulus is from attention-related psychological experiments [14], as detailed in Section 4.1. In the experiments of static saliency detection in natural images, we evaluate the performance by directly comparing the salient regions generated by eleven state-of-the-art approaches with the human-marked salient regions. The test images are from the MSRA database [1], which has about 5,000 images. For each image, Liu *et al.* [1] provided several rectangles to label the salient object. Moreover, Achanta [28] *et al.* chose 1,000 images from the MSRA database [1] to carry on accurate human-marked salient regions, which is the ground truth for us to test a variety of saliency algorithms with objective performance. In addition, we also test our algorithm performance with a dataset provided by Hou [26], because Hou's method is based on the frequency domain. In the experiment of image sequences of dynamic scenes, we use some videos from the BODIDS dataset [44] and the MSRA video saliency dataset [1]. All tests in this section are implemented in MATLAB and performed on the Windows XP platform with Intel Core2 2.2GHz CPU and 2G Memory.

### 4.1. Responses to Psychological Patterns

**Figure 4.**
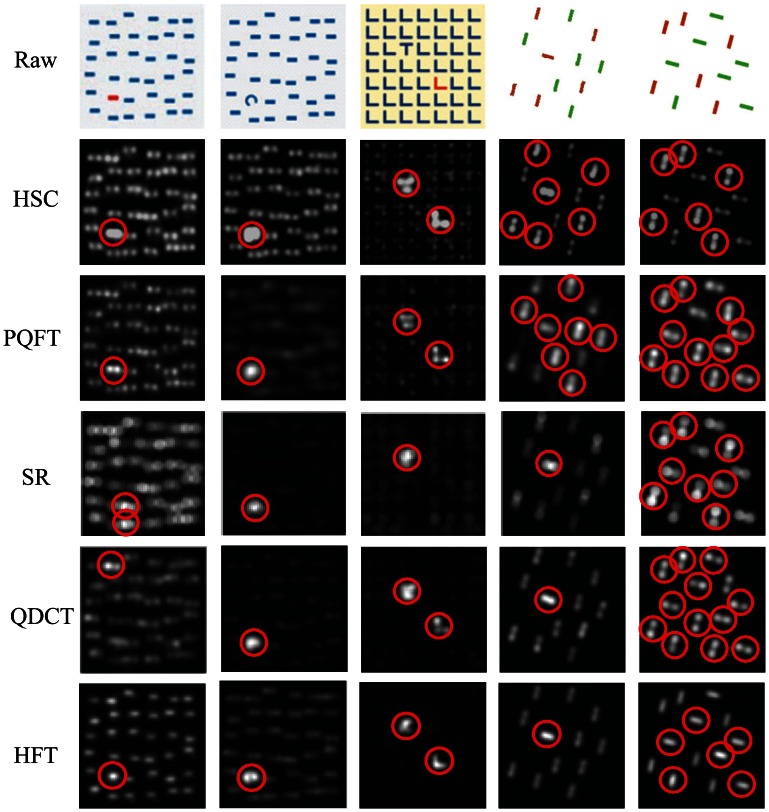
Comparison of our method with [26,27,34,39] on psychological patterns. The first row is the raw images; the second to sixth row are results produced by our method (HSC) and [26,27,34,39], respectively.

Psychological patterns, such as those shown in Figure 4 are widely used in visual attention experiments, not only to explore the mechanism of visual search, but also to test the effectiveness of the saliency map. We test our model on several psychological stimuli that are commonly used to represent pre-attentive visual features and some mixed stimulus [14]. These patterns include “line orientation”, “length”, “size”, “closure”, “curvature”, “density”, “number”, “intersection”, “terminator”, “color” and mixed stimulus, *etc.*

In this experiment, we use five stimulation patterns to test our approach, and our model does not include non-uniform sampling technology for psychological testing of fairness. As shown in Figure 4, we compare our results with four state-of-the-art saliency detection methods related to our approach, which are amplitude spectral residual (SR) [26], phase spectrum of quaternion Fourier transform (PQFT) [27], quaternion discrete cosine transform (QDCT) [39] and optimal scale-space analysis of the hypercomplex Fourier transform (HFT) [34]. In Figure 4, the first column image is a salient color stimulus. Except SR, all of the other methods successfully find the stimulus target. These results shows that the hypercomplex Fourier transform has better results in the color space. The second column gives a salient curvature stimulus. All five methods successfully detect the salient area. These results show that the frequency-based saliency detection methods perform well on salient texture. The third column is a pattern of a combination of stimuli with “intersection” and “color”; our method is stronger in a salient stimulus than the other three methods (PQFT, QDCT and HFT). In this mixture pattern, SR only detected out the “intersection” region and failed to find other “color” regions. In particular, the last two columns are complex stimulus cases, which are stimulus patterns composed of “line orientation” and “color”. The SR and PQFT methods fail to find all red dashes, because they consider amplitude spectral or phase spectral separately. The QDCT and HFT also fail to perceive these red dashes, since they consider the key factor as a local patch spectrum or an optimal scale-space of an image. In contrast, our method has a good performance, because we comprehensively consider the global contrast of amplitude and phase spectral in the saliency detection model.

### 4.2. Static Saliency Detection in Natural Images

In this subsection, we test our method on the salient object detection dataset provided by Achanta [28] and the saliency detection dataset based on frequency domain provided by Hou [26]. These two datasets have 1,062 images with corresponding ground-truth. They cover many different salient objects in different image sizes, such as human, flower, car, bird, house, boat, sportsman, text and sign, in a simple or complex cluttered background. For a fair test, we set the saliency map at the resolution of 320 × 240 in all experiments, then resize it to raw size. For better visualization, a 2D Gaussian filter with *σ* = 3 is performed on all the results. We evaluate and compare our approach with eleven existing methods using qualitative and quantitative performance evaluation, respectively.

**Figure 5.**
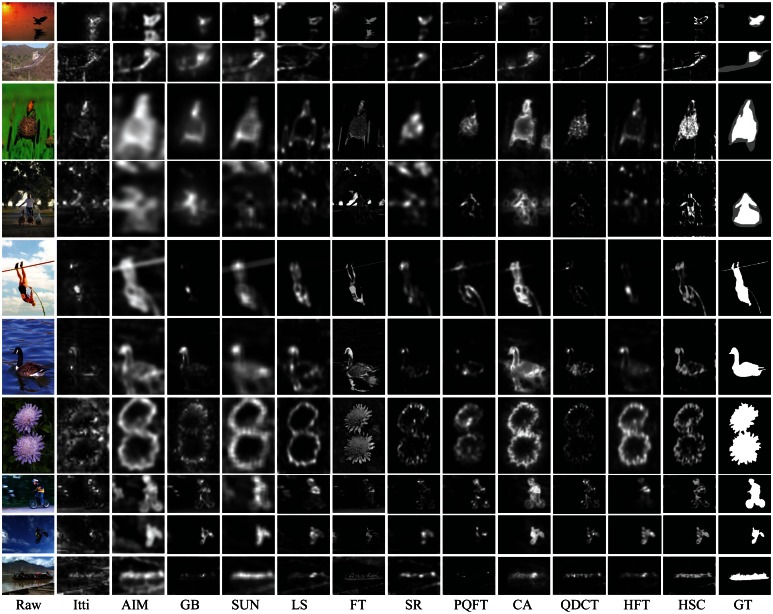
Comparison of our method with eleven state-of-the-art saliency methods. The first column is the raw images (Raw), the last column is the ground truth (GT), the second to twelfth column are results produced by Itti [13], attention information maximization (AIM) [14], graph-based visual saliency (GB) [15], saliency using natural statistics on Bayesian framework (SUN) [16], self-resemblance (LS) [18], FT [28],frequency-tuned approach (FT) [26], PQFT [27], context-aware saliency (CA) [25], QDCT [39], hypercomplex Fourier transform (HFT) [34] and our proposed method (HSC), respectively.

In qualitative comparison, we show our saliency map and compare to the other eleven state-of-the-art algorithms, which are the classic model (Itti) [13], attention information maximization (AIM) [14], graph-based visual saliency (GB) [15], saliency using natural statistics on Bayesian framework (SUN) [16], saliency detection by self-resemblance (LS) [18], frequency-tuned approach (FT) [28], SR method [26], PQFT approach [27], context-aware saliency (CA) [25], QDCT method [39] and HFT approach [34]) in Figure 5. For the Itti *et al.* approach, we used source code from saliencytoolbox of webpage [46], and for N.Bruce's method (AIM), J.Harel's method (GBVS), Zhang's method (SUN), H.Seo's method (LS), R.Achanta's method (FT), Hou's method (SR) and Goferman's method (CA), S.Boris' method (QDCT) and J.Li's method (HFT), we used source code from the authors' website. For Guo's method (PQFT), we implement the method in MATLAB using the “qtfm” toolbox [41], since we could not have access to the author's code. These codes all run on the MATLAB platform.

Although similar to SR and PQFT, which use frequency domain in saliency detection, the proposed method performs better than the two methods, since we consider not only amplitude spectral, but also phase spectral for global contrast in an image. Although the saliency map from [25] is very similar to ours, our method averagely takes about 0.2 s to compute a saliency map, while CA [25] costs 60 s on average, using the same computing conditions. Also, others models' computational time costs are shown in Table 1.

Figure 5 gives the selected results of twelve methods in ten natural images, which shows that our saliency map can more successfully detect the salient birds, Great Wall, girl, flower, sportsman, boat, *etc.*, in each scene than other approaches. However, other methods can detect salient objects or just a part of these objects or almost failed. From Figure 5, it can be observed that the proposed approach is more robust than other models, and the detected saliency region by the proposed method is close to human hand-labeled images (ground truth, GT).

**Figure 6.**
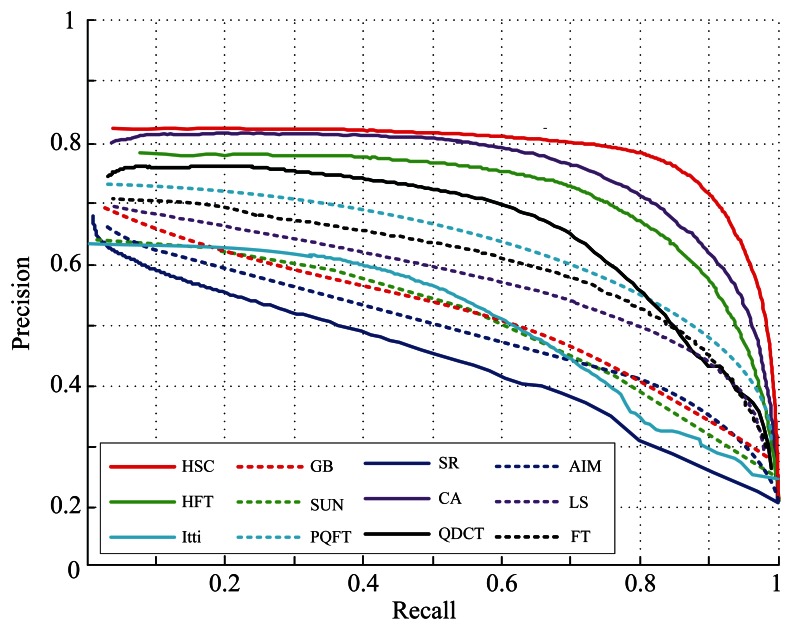
The Precision-recall curve for naive thresholding of saliency maps using 1,000 publicly available benchmark images with our proposed method (HSC) and the other eleven methods (Itti [13], AIM [14], GB [15], SUN [16], LS [18], FT [28], SR [26], PQFT [27], CA [25], QDCT [39] and HFT [34]) in two datasets [1,26].

For quantitative performance evaluation, we compare our model with the above eleven methods using a precision *vs.* recall (PR) measurement introduced in [28]. The saliency map values are in the range of [0, 255]. The simplest way to get a binary segmentation of salient objects is to threshold the saliency map with a threshold in [0, 255]. To compare the quality of different saliency maps, we vary this threshold from 0 to 255 and compute the precision and recall at each value of the threshold. Figure 6 shows the resulting precision vs. recall (PR) curves. The PR curves clearly show that our method performs better than the other eleven methods in human hand-labeled results. At the minimum recall values, the precision of our method is higher than that of the other methods, because the saliency map parallel computed by our method is a global spectral contrast and contains more pixels with the saliency value 255. Meanwhile, as shown in Figure 5, the proposed method also outperforms the other methods in robustness, the integrity of the salient object and consistency to ground-truth data.

### 4.3. Saliency Detection in Dynamic Scenes

**Figure 7.**
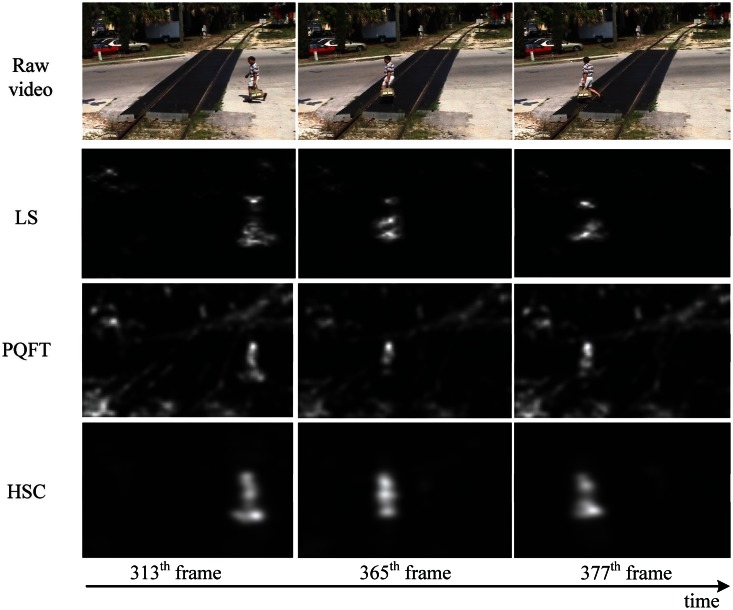
Comparison of two methods of spatio-temporal saliency maps in video sequences (static background). The first row is the raw video [44]; the second to fourth row are results produced by LS [18], PQFT [27] and the proposed method (HSC), respectively.

**Figure 8.**
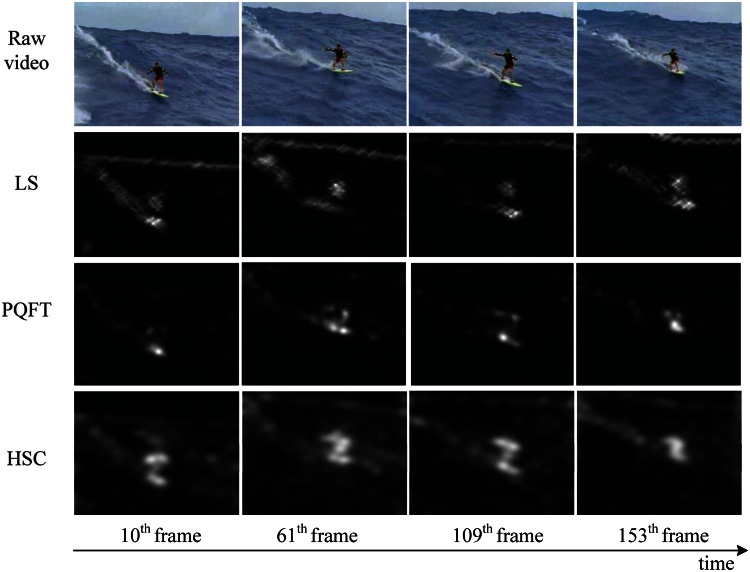
Comparison of two methods of spatio-temporal saliency maps in video sequences (dynamic background). The first row is the raw video [1]; the second to fourth row are results produced by LS [18], PQFT [27] and the proposed method (HSC), respectively.

The video data is generally divided into two kinds, dynamic background and static background. In order to show the performance of the proposed method in spatio-temporal dynamic scenes, we use two type of videos from [44] (static background) and [1] (dynamic background), respectively. For video of static background, such as Figure 7, this group of data are about a railroad intersection taken by a fixed monitor, with an image size of 360 × 240 pixels, 500 frames. For video of dynamic background, such as Figure 8, this group of data are about an athlete surfing on the sea, with an image size of 320 × 240 pixels, 198 frames. Note that we keep the visual resolution of the saliency map to 128 × 128 pixels here in order to save video computational cost. To testify to the efficiency of our spatiotemporal saliency, we compare the proposed HSC method with the LS [18] and PQFT [27] methods in Figure 8. The LS method is a novel bottom-up approach for space-time saliency detection using local regression kernels. However, for the simultaneous movement of the background and foreground object, in particular, the LS method succeeds in capturing highly the textured backgrounds, but fails to detect the motion of objects, such as is shown in Figure 8. Moreover, the proposed method and PQFT all successfully compute the motion object in two types of dynamic scenes, but the proposed method can extract more integrity-salient objects. The receiver operating characteristic (ROC) curve serves as a criterion for performance evaluation. Table 2 lists the ROC areas of different algorithms and shows that our algorithm achieves the highest ROC area. The subjective and objective data show that our approach is more robust than other models in saliency region detection. Thus, our method has a better performance on spatio-temporal saliency detection.

## 5. Discussion

In this section, we discuss the connection and clarify the difference between our method for visual saliency perception and other related methods.

### 5.1. Hypercomplex Frequency Spectral Contrast versus SR and PQFT

The spectral residual (SR) method [26] introduces frequency analysis to visual saliency perception. This work is based on the amplitude spectrum of representative natural image statistics on a single scale of a gray image. Following this, the PQFT method [27,40] claims that the phase spectrum of Fourier transform is important to visual saliency, including the multi-scale case. In this paper, our work is based on the visual perspective of multi-resolution characteristics. We assume that the salient object perception is the accumulation result of the visual saliency in multi-scale spectrum contrast. Therefore, we introduce the blurred image to mimic an image average visual stimulation. By point to point comparison of the raw image and an average energy spectral image, the proposed method can get the saliency stimuli in parallel computing of the hypercomplex Fourier transform. However, unlike PQFT or SR, the information of visual saliency is reconstructed by using the phase spectrum or amplitude spectrum. The objective and subjective experimental results (Figures 5 and 6) show that our proposed method is better than the two algorithms mentioned above.

### 5.2. Hypercomplex Frequency Spectral Contrast versus QDCT and HFT

**Figure 9.**
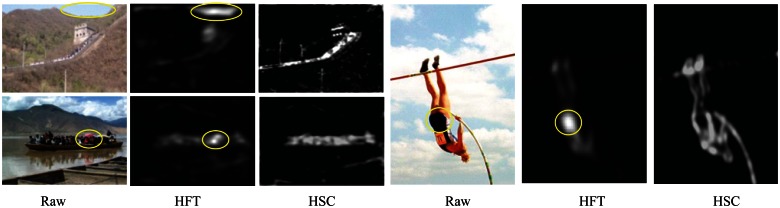
The results of saliency maps using HFT and HSC.

In this paper, visual perception is a bottom-up data-driven computation process. The saliency detection in a bottom-up manner is a global contrast result of features. The QDCT algorithm [39] depends on the division of the image block size. The saliency map has obvious blockiness, as shown in Figure 1(e). The HFT algorithm [34] points out that the maximum of visual saliency stimulation exist in an optimal scale space, while the saliency contribution is weaker in the other scale space. HFT emphasizes the greater importance of local single-scale features more than the global multi-scale features. From Figure 9, we can see that the HFT algorithm was concerned more with the local differences, such as the Great Wall, image of the sky, clothes and other regional players. Although Our algorithm, QDCT and HFT all use the hypercomplex frequency domain in parallel computing, the proposed algorithm emphasizes the global various scales spectrum result's overall role of stimulation on visual saliency.

## 6. Application of Moving Object Extraction

In this section, we extend our approach to applications of moving object extraction to show its useful and plentiful potentials in visual media perception. Moving object detection in complex scenes is an important and challenging problem in computer vision, which is used in many applications, such as video surveillance, object tracking, video content compression and video semantic analysis. The classical method is background modeling. The background subtraction method can be applied under certain assumptions, such as a static background or a fixed camera. However, for dynamic backgrounds, this method is more difficult in detecting the holistic motion of an object. In this subsection, we overcome these limitations using our proposed spatio-temporal saliency method. Moving objects detection can be seen as a spatio-temporal saliency detection problem. From the view of the spatio-temporal saliency feature, moving objects have higher contrast in the frequency domain. The frame difference of a moving object may form the big peak value in the hypercomplex spectrum contrast. Therefore, the proposed method is much easier for detecting the space-time salient moving object by spatio-temporal global features.

**Figure 10.**
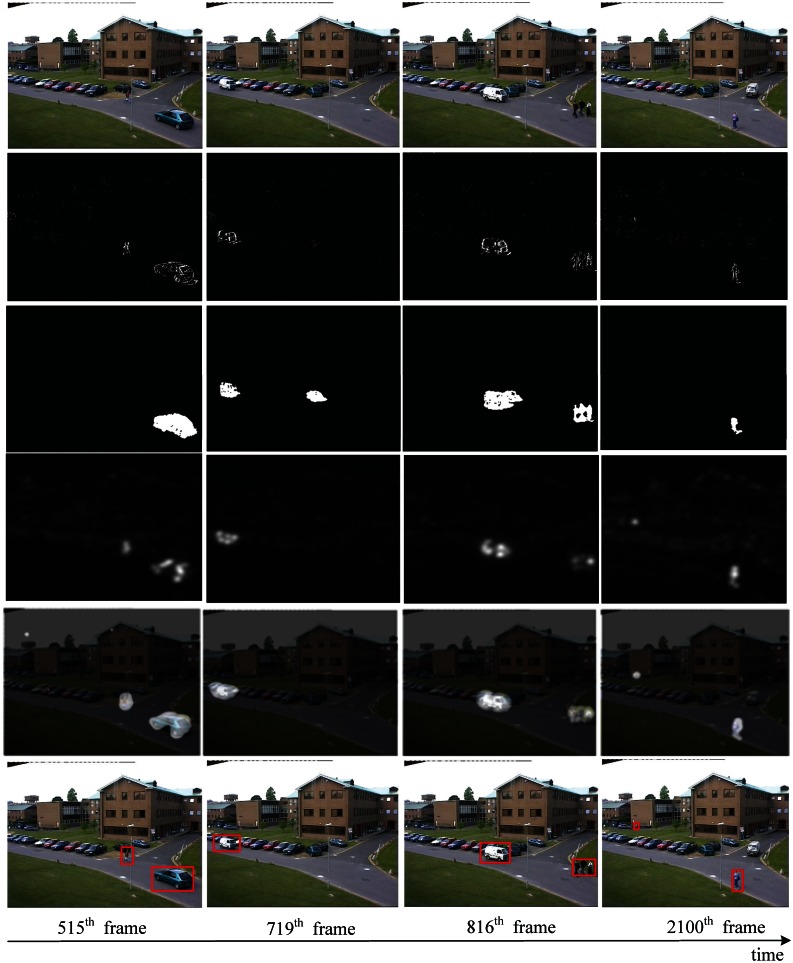
Comparison of two methods of spatio-temporal saliency maps in video sequences. The first row is the raw video of PETS2001 [45]; the second to fourth row are results produced by GMM [47], KDE [48], our method (HSC), mask of moving object extraction results by our method and box label of moving object extraction results by our method, respectively.

**Figure 11.**
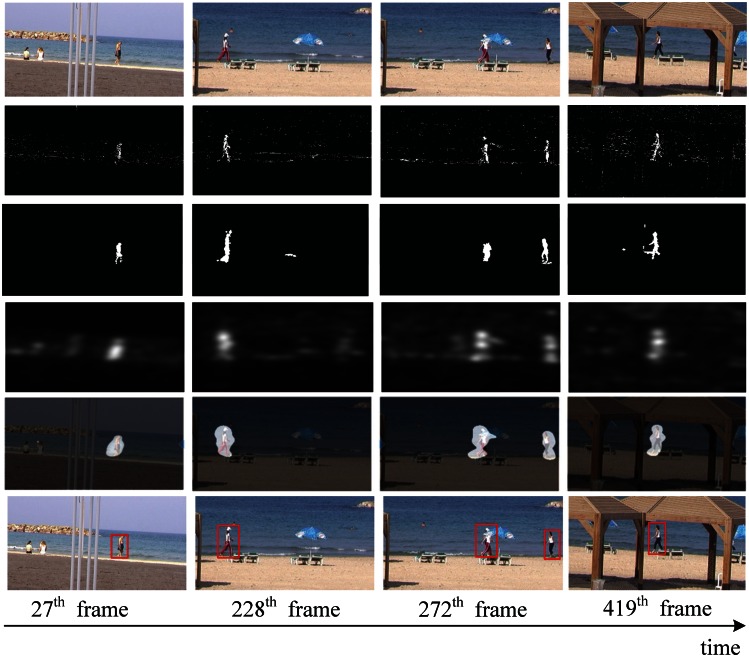
Comparison of two methods of spatio-temporal saliency maps in video sequences. The first row is the raw video of a dynamic background beach video [49]; the second to fourth row are results produced by GMM [47], KDE [48], our method (HSC), mask of moving object extraction results by our method and box label of moving object extraction results by our method, respectively.

For the performance evaluation of moving objects detection, we employ two public datasets, PETS2001 [45], and a beach video [49], which are all taken in the outdoor environment and contain dynamic illumination changes. The image resolution of each dataset is 768 × 576 pixels (2,100 frames) and 360 × 180 pixels (457 frames). In the beach video, there are multiple people (foreground) walking through the beach with moving waves (background). We compare the proposed saliency approach with two methods, the traditional GMM [47] and KDE [48] algorithm, which are widely used for motion detection. GMM is a classic, probabilistic method for background subtraction. But for both the dynamic background and dynamic foreground object, in particular, the movement examination ability of GMM is limited, because of the noise and dynamic background. For the time cost, the GMM method is more time-consuming. Similarly, KDE is a classical algorithm for moving object segmentation. Using a few frames as *priori* knowledge, the KDE method can model the background and quickly extract the moving targets in subsequent frames. However, the KDE method is sensitive to illumination changes, and some small moving objects are easily lost using KDE. The results are shown in Figures 10 and 11. Finally, our proposed moving object detection method can give good performance with a simple binary threshold and morphological operators in our proposed saliency map. For the quantitative comparison, the recall and precision defined employ H. Seo's method [18] to determine a threshold value efficiently. The bounding boxes of moving objects are used as the ground-truth. If at least 30% of pixels within each bounding box are classified as foreground pixels, it can be easily detected as the moving object by using the simple post processing. We compare HSC with other approaches based on this recall rate. The recall and precision values computed from 20 frames randomly taken throughout the entire dataset are shown in Table 3. Note that the low precision value indicates that false ones occur more frequently. From these test results, we confirm that the proposed approach can be effectively employed for extracting moving objects.

## 7. Conclusions

In this paper, we presented a spatio-temporal saliency perception method inspired by hypercomplex spectrum contrast and human visual perception. The basic idea is that the salient object is highly sensitive to the contrast of integrated features and nonlinear non-uniform sampling of visual information. To this end, a novel hypercomplex spectrum method for spatiotemporal saliency detection has been designed. The hypercomplex amplitude spectrum represents the power of the intensity, color, motion features and the hypercomplex phase spectrum to represent the texture and location information. We use the original image hypercomplex spectrum comparison with the down-sampling image spectrum to pop out the salient region and use non-uniform sampling to be consistent with the human visual perception of salient regions. The proposed method is able to effectively and quickly detect salient regions from an image and give better responses to psychological patterns. Experimental results show that the proposed method has better performance compared with the other eleven state-of-the-art methods on two public static image datasets. In addition, we applied the proposed method to image auto-segmentation and moving salient object detection. Since there is natural integration of various visual features in the hypercomplex spectrum-domain, the proposed method can efficiently detect the initial segmentation area and moving objects in cluster static or dynamic scenes. Experimental results show that our method has plentiful possibilities to some promising applications in image or video perception processing.

## Figures and Tables

**Table 1.** Average time cost to compute a saliency map for an image in two datasets [1,26].

Method	IT [13]	AIM [14]	GB [15]	SUN [16]	LS [18]	FT [28]
Time(s)	0.923	5.264	2.875	3.314	6.782	0.027
Method	SR [26]	PQFT [27]	CA [25]	QDCT [39]	HFT [34]	HSC
Time(s)	0.087	0.121	60.3	0.163	0.197	0.209

**Table 2.** receiver operating characteristic (ROC) areas of different approaches.

**Type of Video**	**Static Background**			**Dynamic Background**		
Approaches	LS [18]	PQFT [27]	HSC	LS [18]	PQFT [27]	HSC
ROC area	0.7015	0.6823	0.7302	0.6321	0.6118	0.6527

**Table 3.** Peformance evaluation for moving object extraction.

**Video dataset**	**PETS2001**			**Dynamic background**		
Approaches	GMM [47]	KDE [48]	HSC	GMM [47]	KDE [48]	HSC
Recall	0.3132	0.3627	0.3729	0.3067	0.3541	0.3211
Precision	0.5231	0.8014	0.7916	0.3231	0.4014	0.6835

## References

[1] Liu T., Yuan Z., Sun J., Wang J., Zheng N., Tang X., Shum H. (2011). Learning to detect a salient object. IEEE Trans. Pattern Anal. Mach. Intell..

[2] Wang T., Zheng N., Xin J., Ma Z. (2011). Integrating millimeter wave radar with a monocular vision sensor for on-road obstacle detection applications. Sensors.

[3] Rokszin A., Márkus Z., Braunitzer G., Berényi A., Benedek G., Nagy A. (2010). Visual pathways serving motion detection in the mammalian brain. Sensors.

[4] Fu Y., Cheng J., Li Z., Lu H. Saliency Cuts: An Automatic Approach to Object Segmentation.

[5] Rahtu E., Kannala J., Salo M., Heikkilä J. Segmenting Salient Objects from Images and Videos.

[6] Siagian C., Itti L. (2007). Rapid biologically-inspired scene classification using features shared with visual attention. IEEE Trans. Pattern Anal. Mach. Intell.

[7] Hampapur A., Brown L., Connell J., Ekin A., Haas N., Lu M., Merkl H., Pankanti S. (2005). Smart video surveillance: Exploring the concept of multiscale spatiotemporal tracking. IEEE Signal Process. Mag..

[8] Walther D., Itti L., Riesenhuber M., Poggio T., Koch C. Attentional Selection for Object Recognition: A Gentle Way.

[9] Xue J., Li C., Zheng N. (2011). Proto-object based rate control for JPEG2000: An approach to content-based scalability. IEEE Trans. Image Process.

[10] Deco G., Zihl J. (2001). Top-down selective visual attention: A neurodynamical approach. Visual Cogn..

[11] Oliva A., Torralba A., Castelhano M., Henderson J. Top-down Control of Visual Attention in Object Detection.

[12] Judd T., Ehinger K., Durand F., Torralba A. Learning to Predict Where Humans Look.

[13] Itti L., Koch C., Niebur E. (1998). A model of saliency-based visual attention for rapid scene analysis. IEEE Trans. Pattern Anal. Mach. Intell.

[14] Bruce N., Tsotsos J. Saliency Based on Information Maximization.

[15] Harel J., Koch C., Perona P. Graph-Based Visual Saliency.

[16] Zhang L., Tong M., Marks T., Shan H., Cottrell G. (2008). Sun: A bayesian framework for saliency using natural statistics. J. Vis..

[17] Gao D., Mahadevan V., Vasconcelos N. (2008). On the plausibility of the discriminant center-surround hypothesis for visual saliency. J. Vis..

[18] Seo H., Milanfar P. (2009). Static and space-time visual saliency detection by self-resemblance. J. Vis..

[19] Rapantzikos K., Tsapatsoulis N., Avrithis Y., Kollias S. (2009). Spatiotemporal saliency for video classification. Signal Process. Image Commun..

[20] Gopalakrishnan V., Hu Y., Rajan D. (2009). Salient region detection by modeling distributions of color and orientation. IEEE Trans. Multimed..

[21] Zhang L., Tong M., Cottrell G. Sunday: Saliency Using Natural Statistics for Dynamic Analysis of Scenes.

[22] Mahadevan V., Vasconcelos N. (2010). Spatiotemporal saliency in dynamic scenes. IEEE Trans. Pattern Anal. Mach. Intell.

[23] Cheng M., Zhang G., Mitra N., Huang X., Hu S. Global Contrast Based Salient Region Detection.

[24] Li C., Xue J., Zheng N., Tian Z. Nonparametric Bottom-up Saliency Detection Using Hypercomplex Spectral Contrast.

[25] Goferman S., Zelnik-Manor L., Tal A. Context-aware Saliency Detection.

[26] Hou X., Zhang L. Saliency Detection: A Spectral Residual Approach.

[27] Guo C., Ma Q., Zhang L. Spatio-temporal Saliency Detection Using Phase Spectrum of Quaternion Fourier Transform.

[28] Achanta R., Hemami S., Estrada F., Susstrunk S. Frequency-tuned Salient Region Detection.

[29] Treisman A., Gelade G. (1980). A feature-integration theory of attention. Cogn. Psychol.

[30] Koch C., Poggio T. (1999). Predicting the visual world: Silence is golden. Nature.

[31] Piotrowski L., Campbell F. (1982). A demonstration of the visual importance and flexibility of spatial-frequency amplitude and phase. Perception.

[32] Oppenheim A., Lim J. (1981). The importance of phase in signals. Proc. IEEE.

[33] Li J., Levine M., An X., He H. Saliency Detection Based on Frequency and Spatial Domain Analyses.

[34] Li J., Levine M., An X., Xu X., He H. (2013). Visual saliency based on scale-space analysis in the frequency domain. IEEE Trans. Pattern Anal. Mach. Intell.

[35] Fang Y., Lin W., Lee B., Lau C., Chen Z., Lin C. (2012). Bottom-up saliency detection model based on human visual sensitivity and amplitude spectrum. IEEE Trans. Multimed..

[36] Yu Y., Wang B., Zhang L. (2011). Bottom–up attention: Pulsed pca transform and pulsed cosine transform. Cogn. Neurodynamics.

[37] Hou X., Harel J., Koch C. (2012). Image signature: Highlighting sparse salient regions. IEEE Trans. Pattern Anal. Mach. Intell.

[38] Boris S., Stiefelhagen R. Predicting Human Gaze Using Quaternion DCT Image Signature Saliency and Face Detection.

[39] Boris S., Stiefelhagen R. Quaternion-Based Spectral Saliency Detection for Eye Fixation Prediction.

[40] Guo C., Zhang L. (2010). A novel multiresolution spatiotemporal saliency detection model and its applications in image and video compression. IEEE Trans. Image Process.

[41] Ell T., Sangwine S. (2007). Hypercomplex Fourier transforms of color images. IEEE Trans. Image Process.

[42] Kim W., Jung C., Kim C. (2011). Spatiotemporal saliency detection and its applications in static and dynamic scenes. IEEE Trans. Circ. Syst. Video Technol..

[43] Zhai Y., Shah M. Visual Attention Detection in Video Sequences Using Spatiotemporal Cues.

[44] Sheikh Y., Shah M. (2005). Bayesian modeling of dynamic scenes for object detection. IEEE Trans. Pattern Anal. Mach. Intell.

[45] Ferryman J. Pets'2001 database. http://www.cvg.cs.rdg.ac.uk/PETS2001/pets2001-dataset.html.

[46] Walther D., Itti L. SaliencyToolbox homepage. http://www.saliencytoolbox.net/.

[47] Stauffer C., Grimson W. Adaptive Background Mixture Models for Real-Time Tracking.

[48] Elgammal A., Duraiswami R., Harwood D., Davis L. (2002). Background and foreground modeling using nonparametric kernel density estimation for visual surveillance. Proc. IEEE.

[49] Shechtman E., Irani M. Space-time Behavior Based Correlation.

